# A new electronic valve for the treatment of NPH

**DOI:** 10.1186/2045-8118-12-S1-O25

**Published:** 2015-09-18

**Authors:** Christoph Miethke, Joerg Binkele, Thoralf Knitter

**Affiliations:** 1 Christoph Miethke GmbH & Co KG, Germany

## 

The first shunts being successfully used for the treatment of hydrocephalus were designed to lower an overpressure within the ventricles of the brain. Later the same technology was used for the treatment of normal pressure hydrocephalus, although the background of this disease can not be compared with obstructive hydrocephalus. Since then no specific device has been developed focussing on the treatment of NPH. However, the recent knowledge of NPH and the risks behind the shunting of NPH offer interesting options.

For the diagnosis of NPH an often used method is the spinal tap test. The withdrawal of up to 50 cc of spinal fluid sometime leeds to impressive clinical improvement. To address this fact a new device has been developed which can be programmed to open depending on time and body position. Consequently in contrast to valves for the treatment of obstructive hydrocephalus this device does not open depending on increased baseline ICP or ICP peaks. The device can be programmed to open for two minutes intervals over 24 hours. The valve can be programmed to remain closed, to open for two minutes within 24 hours, to open the whole 24 hours or any time between two minutes and 24 hours. In addition it can be programmed whether or not the opening should be manipulated by the body position of the patient in particular to allow an only reduced flow in the standing position.

The new electronic device is the first implant developed only for NPH addressing specific aspects of this decease. The valve has been investigated in vitro simulating the condition within a patient. It has not been implanted yet, because of regulatory issues. Whether or not the device improved the clinical outcome in patients with NPH, whether or not it can lower risks for these patients and introduce new treatment options can not be answered without clinical experience. However, it promises to be a tool for scientific research, which might help to improve the understanding of NPH.
